# Food-Related Odors Activate Dopaminergic Brain Areas

**DOI:** 10.3389/fnhum.2017.00625

**Published:** 2017-12-19

**Authors:** Agnieszka Sorokowska, Katherina Schoen, Cornelia Hummel, Pengfei Han, Jonathan Warr, Thomas Hummel

**Affiliations:** ^1^Smell & Taste Clinic, Department of Otorhinolaryngology, Technische Universität Dresden, Dresden, Germany; ^2^Smell & Taste Research Lab, Institute of Psychology, University of Wroclaw, Wroclaw, Poland; ^3^Takasago Europe Perfumery Laboratory SARL, Paris, France

**Keywords:** olfaction, edibility, food, fMRI, reward circuit

## Abstract

Food-associated cues of different sensory categories have often been shown to be a potent elicitor of cerebral activity in brain reward circuits. Smells influence and modify the hedonic qualities of eating experience, and in contrast to smells not associated with food, perception of food-associated odors may activate dopaminergic brain areas. In this study, we aimed to verify previous findings related to the rewarding value of food-associated odors by means of an fMRI design involving carefully preselected odors of edible and non-edible substances. We compared activations generated by three food and three non-food odorants matching in terms of intensity, pleasantness and trigeminal qualities. We observed that for our mixed sample of 30 hungry and satiated participants, food odors generated significantly higher activation in the anterior cingulate cortex (right and left), insula (right), and putamen (right) than non-food odors. Among hungry subjects, regardless of the odor type, we found significant activation in the ventral tegmental area in response to olfactory stimulation. As our stimuli were matched in terms of various perceptual qualities, this result suggests that edibility of an odor source indeed generates specific activation in dopaminergic brain areas.

## Introduction

Eating is an essential source of pleasure in human life. Stimulation with food-associated cues of different sensory categories has often been shown to be a potent elicitor of cerebral activity in brain reward circuits, including frontal, ventral striatal, amygdala, and midbrain regions (Berridge, 1996; Beaver et al., 2006; Stoeckel et al., 2008). As palatability tends to be positively correlated with energy density (Drewnowski, 1998), the rewarding aspects of intake of foods high in fat and sugar are probably a dietary legacy from our evolutionary ancestors (Lieberman, 2003), for whom consumption of such products was associated with increased chances for survival.

Among different elements contributing to the rewarding outcome of food ingestion (Rolls, 2015), smells influence and modify the hedonic qualities of eating experience (Schifferstein and Verlegh, 1996; Stevenson, 2010). Studies conducted among hungry individuals show that edibility of odor source is an important determinant of magnitude (Bragulat et al., 2010) and speed of cerebral processing (Boesveldt et al., 2010). In the context of reward processing, compared to non-food odors, under fasting conditions, food odors were shown to evoke higher activation in, e.g., ventral tegmental area (VTA), ventral striatum, and medial frontal cortex (Bragulat et al., 2010), left lateral orbitofrontal cortex and inferior insula (Eiler et al., 2012). Similarly, a mixed-design study involving hungry and satiated participants showed activation in anterior cingulate cortex (ACC) and nucleus accumbens (Nac) in reaction to smells of edible products (Jiang et al., 2015). Food odors elicit strong reactions also among non-fasting individuals. For example, Frasnelli et al. (2015) observed activations in left post-central gyrus, left superior frontal gyrus, and in midbrain (consistent with the ventral tegmental area) for food, as opposed to non-food odors. In another study, odor of chocolate (but not three other control odors) elicited activation in medial frontal cortex (Small et al., 2005).

Overall, different studies suggest that in contrast to smells of non-edible items, perception of food-associated odors may activate dopaminergic brain areas. However, there are certain issues related to the aforementioned studies rendering definite conclusions about the reward value of food vs. non-food odors difficult. These involve the selection of odorants and several variables either not taken into consideration, or not well enough controlled in previous studies. In the current approach, we aimed to address these difficulties.

In the group of odor-related issues, the previously observed effects might have been specific to fruit odors, as analyzed by Frasnelli et al. (2015). Furthermore, as the food odors in that study were on average rated as significantly more intense than the additionally used flower odors, the authors limited their detailed food vs. non-food smells comparison to strawberry and lavender which were matched in terms of intensity and pleasantness. To complement the findings of this paper, we decided not to restrict the selection of food-associated odors to fruit smells, but to include different (pleasant) food-associated stimuli, i.e., chocolate, cinnamon and vanilla. In addition, we carefully adjusted the concentrations of both groups of applied odorants to obtain perceptually similar intensities. Finally, we assessed whether participants felt trigeminal in addition to olfactory sensations. Subjects also rated the perceived smelled sweetness of the stimuli, as sweetness of food-related odors could be related to activation of left insula (Bragulat et al., 2010).

As obese and normal-weight individuals may differ in terms of activations of reward areas due to food-related stimuli (Stoeckel et al., 2008; Pursey et al., 2014; Jiang et al., 2015) and—conversely, differences in reward processing can lead to obesity (Stice et al., 2013) or eating disorders (DiLeone et al., 2012), we focused on normal-weight participants. Finally, as hunger might modify the neural activation during stimulation with food odors (Bragulat et al., 2010; Jiang et al., 2015), we assessed subjects' state of hunger. To maintain ecological validity, however, we did not require participants to fast or to eat beyond satiety.

In summary, in the current study we aimed to verify previous findings related to the rewarding value of food-associated odors involving carefully selected odors of edible and non-edible substances.

## Methods

### Participants

Thirty healthy volunteers participated in the study: 17 females aged 22–28 years (24.47 ± 1.84 years) with BMI between 18.78 and 23.41 (20.98 ± 1.26) and 13 males aged 22–29 years (25.08 ±2.29 years) with BMI between 20.01 and 29.39 (23.94 ± 2.4). Two male participants were excluded from further analyses due to overweight (BMI > 25), as being overweight may bias the reward processing of odors (Jiang et al., 2015). Participants were recruited on the campus of the University of Dresden Medical School. They received a moderate sum for participating in the study. Normosmia was ascertained by the shortened, 12-item version of the “Sniffin' Sticks” (Hummel et al., 1997, 2007) identification subtest (Burghart Messtechnik, Wedel, Germany). The Edinburgh Handedness Inventory (Oldfield, 1971) was applied to include right-handed individuals only. The study was conducted according to the Declaration of Helsinki and was approved by the Ethics Board of the University of Dresden Medical School (application number EK 355092013). Participants provided written informed consent prior to study inclusion.

### Odors

Participants were stimulated with six odors: three food related odors (FO) including chocolate (Ch; product code JSEDIB017CK), vanilla (V; product code DGFLO908B), cinnamon (Ci; product code DGWOOD055), and three non-food related odors (NFO) including lily of the valley (Li; product code DGFLO793A), jasmin (J; product code DGFLO770G), and lavender (La; product code DGFLO794). All odorants were provided by Takasago, Paris, France. In order to obtain stimuli of similar intensities, we prepared dilutions of the odorants with dipropylene glycol [product code CAS 25265-71-8; concentrations between 1:1 and 1:300; Ch: 0.5/60 ml (1:120); V: 50/50 ml (1:1); Ci 0.5/150 ml (1:300); Li 0.5/75 ml (1:150); J: 0.5/75 ml (1:150); La: 0.5/80 ml (1:160)]. Similarity of intensities was verified in a pre-test involving 10 subjects, and perceived edibility of FO and NFO was assessed in another pretest, involving 11 subjects. Odor stimulation was performed using a dedicated olfactometer (Sommer et al., 2012), allowing for computer controlled odor delivery with steep stimulus onset and offset within the scanner. Odors were delivered with a flow rate of 1 L/min per nostril.

### Imaging procedures and behavioral measures

For the fMRI sessions, a block design was used with each block consisting of one 20-s “on” period and one 20-s “off” period. Blocks were repeated 6 times for each odorant, thus constituting 6 sessions of 6 min duration each. To avoid habituation, during “on” blocks odors were delivered in pulses of 1 s, with 2 s without odorant between two pulses. During “off” blocks, air flow was switched off. The order of presentation of the odors was pseudorandomized—we created 6 different sequences of odorants. After the scanning session for each odor, subjects orally rated the respective odor according to previously introduced scales via the intercom system. Participants evaluated odors with respect to 5 dimensions: pleasantness (−5 to +5), familiarity, trigeminal sensations (such as tickling, cooling, stinging), intensity, and sweetness (0–10 each). The anchors for pleasantness ratings were “very unpleasant” and “very pleasant”; for familiarity “completely unfamiliar” and “extremely familiar”; for trigeminal sensations ratings, “no additional trigeminal sensations” and “extreme additional trigeminal sensations”; for intensity, “not perceivable” and “extremely intense”; and for sweetness ratings, “not sweet at all” and “very sweet.” Duration of the entire scanning procedure was approximately 60 min. After its completion participants were asked to rate their present state of hunger (from 0 = not hungry at all to 10 = very hungry). Subjects were divided into 3 groups according to their self-assessed level of hunger: a group of hungry subjects (HU, *n* = 10) was defined according to their hunger scores between 7 and 10; a neutral group with hunger ratings ranging between 3 and 6 (NETHU, *n* = 9), and a non-hungry group (NOHU, *n* = 9) with hunger scores between 0 and 2. Only the hungry and non-hungry subsamples were further analyzed in detail.

All participants attended a mock scanning session before the test, where they were familiarized with the odors, stimulation procedure and rating scales. Further, subjects trained a breathing technique called velopharyngeal closure in order to prevent sniffing in response to odorous stimulation during scanning. This method is commonly applied in fMRI (Small et al., 2005; Bensafi et al., 2012; Sorokowska et al., 2016) and EEG studies (Iannilli et al., 2015) on olfactory perception.

### Imaging data acquisition

Trained technicians acquired functional images on a 1.5 T scanner (Siemens Sonata) with the following parameters: EPI (echo planar imaging) sequence, Repetition Time: 2,500 ms, Echo Time: 40 ms; Flip Angle: 90°, Matrix: 64 × 64, voxel size: 3 × 3 × 3.75 mm. For overlays we also acquired anatomical T1 scans (magnet prepared rapid gradient echo: MP-RAGE; Repetition Time: 2,180 ms, Echo Time: 3.93 ms, Flip Angle: 15 degrees, Matrix 352 × 384, voxel size: 1 × 0.73 × 0.73 mm).

### Imaging data analyses

Neuroimaging data were analyzed by means of SPM 12 (Wellcome, London, UK) within the MatLab framework (MathWorks, Ismaning, Germany). Pre-processing steps included realignment and unwarping, co-registration of functional with anatomical images, segmentation into compartments of white and gray matters and cerebro-spinal fluid, smoothing (with full width at half maximum of the Gaussian kernel of 7 mm in each direction) and normalization with respect to the Montreal Neurological Institute (MNI) space.

Using the canonical hemodynamic function and its derivative set available in SPM12, the pre-processed functional data for the single subject analyses were inserted into six separate general linear models for the six odors, each including two experimental conditions (odor ON period, odor OFF period) and movement parameters. Analyses of pooled odors were also performed for individuals, with all six odors as an entire group, and with odors grouped according to their food/non-food characteristics. The following contrasts were modeled at individual level: contrast for individual odor (1 −1), contrast for FO (1 −1 1 −1 1 −1 0 0 0 0 0 0), contrast for NFO (0 0 0 0 0 0 1 −1 1 −1 1 −1), and contrast for Pooled six Odors (1 −1 1 −1 1 −1 1 −1 1 −1 1 −1). At group level, contrasts from single subjects were used to test brain activation by odors. To test the differences of brain activation between FO and NFO, contrasts for FO and NFO from single subjects were analyzed with ANOVA in SPM, with sweetness, familiarity and hunger ratings as covariates of no interest. In addition, a 2 × 2 full factorial design was built including hunger status (2 levels) and odor type (2 levels) to explore the effect of hunger on cerebral activation of food and non-food odors. Region of interest (ROI) analyses were applied focusing on the reward circuits (including ventral striatum and ventral tegmental area, VTA) and olfactory regions including piriform cortex, amygdala, insula, olfactory orbitofrontal cortex OFC, and anterior cingulate. The ROI for olfactory OFC was based on a 10-mm sphere centered on the right (x, y, z: 24, 36, −12) or left putative olfactory OFC (x, y, z: −22, 32, −16) (Gottfried and Zald, 2005). The VTA ROI was built based on a 6-mm sphere centered at MNI peak coordinates (x, y, z: 6, −10, −12), based on a study showing VTA activation in response to alcohol odors (Kareken et al., 2004). The other masks were created using the “automated anatomical labeling (aal)” atlas (Tzourio-Mazoyer et al., 2002), embedded in the WFU PickAtlas 2.4 software (ANSIR, Wake Forest University, Winston-Salem, NC, USA) (Maldjian et al., 2003). Small volume correction (SVC) in the ROIs was applied, correcting for multiple comparisons (*p* < 0.05, family-wise error rate [FWE] corrected). The underlying signal within the resulting regional areas was extracted for each subject using the MarsBaR toolbox (http://marsbar.sourceforge.net; Brett et al., 2002) and was used for further analysis or display. For display purposes, activations are shown at the initial threshold.

### Analyses of behavioral measures

Statistical analyses were performed using SPSS 21 (SPSS Inc., Chicago, Ill., USA). Behavioral ratings were compared between FO and NFO using paired *t*-tests, and two sample *t*-tests were applied to compare the estimates for FO and NFO between HU and NOHU groups. Significance level was set at *p* < 0.05 for all analyses.

## Results

### Behavioral data

**Figure 1:**
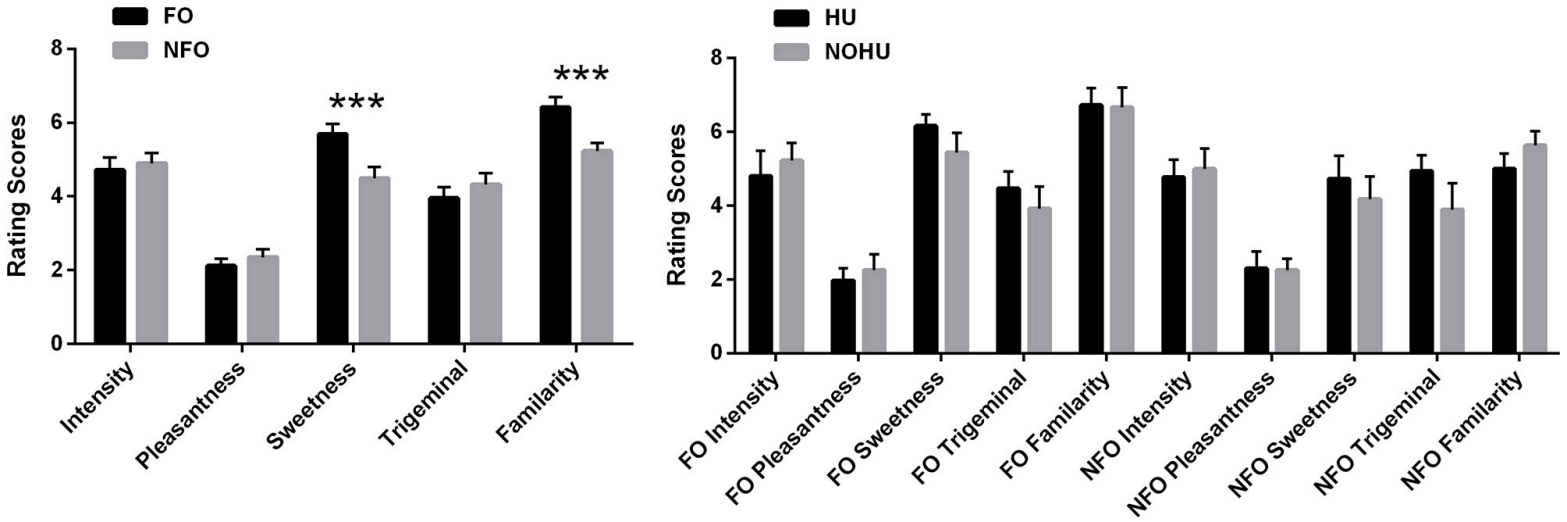
Psychophysical ratings of odors (means and standard error of the mean): **(A)** FO and NFO; **(B)** HU (*n* = 10) and NOHU (*n* = 9) groups. FO, Food Odors; NFO, Non-food Odors; HU, Hunger group; NOHU, Non-hunger group. Asterisks indicate significant differences: ^***^p < 0.001.

Paired *t*-tests for food vs. non-food odors assessments showed that food and non-food odors did not differ significantly in terms of pleasantness [FO: 2.12 ± 1.05, NFO: 2.36 ± 1.11, *t*_(1, 29)_ = −1.22, *p* = 0.23], intensity [FO: 4.71 ± 1.82 NFO: 4.90 ± 1.47, *t*_(1, 27)_ = −0.74, *p* = 0.47], and trigeminal qualities [FO 3.95 ± 1.62 and NFO 4.32 ± 1.62, *t*_(1, 27)_ = −2.00, *p* = 0.06]. However, the perceived sweetness and familiarity of odors differed, with food odors being estimated as sweeter (5.69 ± 1.44) than non-food odors (4.49 ± 1.64) [*t*_(1, 27)_ = 3.70, *p* < 0.001], and more familiar (6.41 ± 1.48) than non-food odors (5.23 ± 1.18) [*t*_(1, 27)_ = 4.55, *p* < 0.001] (Figure 1A). When comparing the HU vs. NOHU groups, behavioral ratings for food and non-food odors did not differ significantly in any aspect (*p* > 0.05, Figure 1B).

### Imaging data

#### Pooled odors ON vs. OFF

First, we compared all odors to baseline (Odors ON-OFF). Whole brain analyses revealed activation in the secondary olfactory areas (insula, Seubert et al., 2013; Fjaeldstad et al., 2017) and reward areas (Putamen). See Table 1 for an overview.

**Table 1:** Brain activation of odor stimulation (contrast all six odors vs. baseline).

	**MNI coordinates**			**Cluster size**	**Peak *T*-value**	**Peak *p*(unc.)**
**Region**	***x***	***y***	***z***			
L Mid Front Gyrus	−20	0	34	42	4.10	0.000
R Putamen	26	20	8	62	4.07	0.000
R Pallidum	18	4	−2	64	3.83	0.000
L Insula	−28	28	16	22	3.74	0.000
R Mid Front Gyrus	42	50	12	20	3.22	0.002
L Sup Front Gyrus	−26	42	0	18	3.12	0.002
	−30	36	4		2.86	0.004
L Insula	−40	2	−6	14	3.12	0.002

*Thresholded at uncorrected p < 0.005, cluster size ≥ 10 voxels, R, right; L, left*.

#### Comparisons of food odors and non-food odors

In the entire sample, using the a priori defined olfactory and reward ROIs, significant brain activation in response to FO vs. NFO was found in the ACC, insula, and putamen (ventral striatum), controlling for the effect of hunger, sweetness, and familiarity (see Table 2). No activation was observed for NFO vs. FO contrast.

**Table 2:** Food odors vs. Non-food odors in all subjects.

**ROI**	**Region**	**MNI coordinates**			**Cluster size**	**Peak *T*-value**	**Peak *p*(unc.)**
		***x***	***y***	***z***			
Olfactory	R ACC^*^	8	46	20	291	4.19	0.000
		14	40	8		4.14	0.000
	L ACC^*^	−4	52	12	238	4.30	0.000
	R Insula	32	−20	20	15	3.16	0.002

*Thresholded at uncorrected p < 0.005, cluster size ≥10 voxels, R, right; L, left; ACC, Anterior Cingulate Cortex*.

* *FWE corrected p < 0.05 with ROI analyses*.

#### Effect of hunger

A full factorial design including hunger state (HU and NOHU) and odor type (FO and NFO) was used to test the effect of hunger, effect of odor type, and the interactive effect between hunger state and odor type on brain activation.

**Figure 2:**
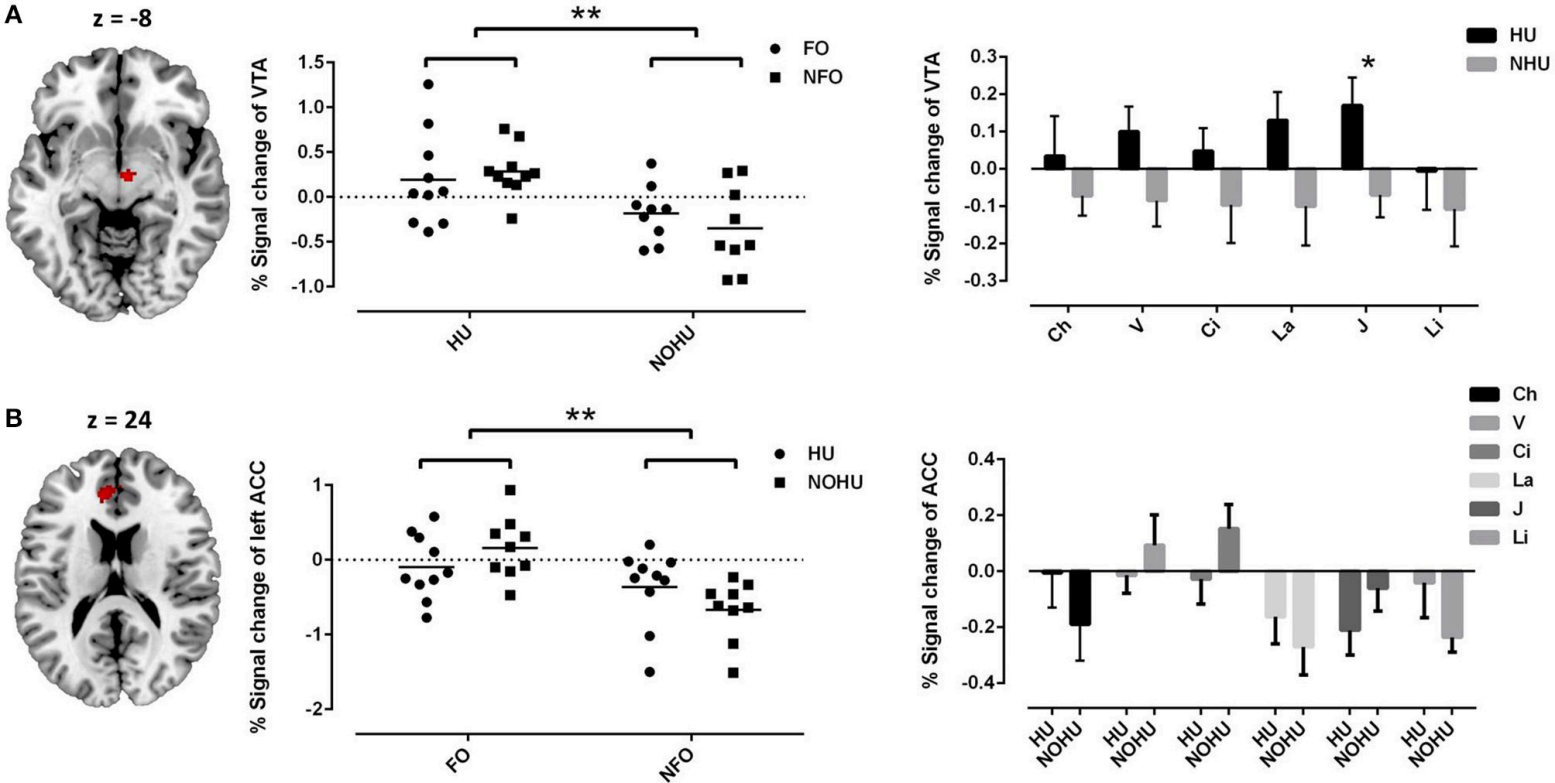
**(A)** VTA activation represents the main effect of hunger; The mean signal change for FO or NFO group and for individual odors of HU and NOHU participants are shown as a scatter plot (individual data and mean) and bar chart (mean and standard error of the mean), separately; **(B)** left ACC activation represents the main effect of odor type (FO vs. NFO). The mean signal change for FO or NFO group and for individual odors rated by HU and NOHU participants are shown as the scatter plot (individual data and mean) and bar chart (mean and standard error of the mean), separately; ACC, anterior cingulate cortex; VTA, ventral tegmental area. FO, Food Odors; NFO, Non-food Odors; HU, Hunger group; NOHU, Non-hunger group; V, Vanilla; Ci, Cinnamon; La, Lavender; J, Jasmine; Li, Lily of the Valley. Asterisks indicate significant differences: ^*^p < 0.05; ^**^p < 0.01.

As the main effect of hunger, we found activation in the VTA (peak at 6 −12 −8, cluster = 22 voxels, Peak T = 4.13, *p*FWEcorr = 0.003, Figure 2A), indicating that hunger increased the reward value of both FO and NFO. Main effect of odor type (FO vs. NFO) showed significant activation in the left ACC (peak at −8 40 18, cluster = 144 voxels, Peak T = 3.89, *p*FWEcorr = 0.049, Figure 2B) and marginally significant activation in the right ACC (peak at 4 42 24, cluster = 41 voxels, Peak T = 3.70, *p*FWEcorr = 0.067). Activation signals within the significant clusters (VTA and left ACC) were extracted and plotted as shown in Figure 2.

No significant interaction of hunger state and odor type was observed in the predicted areas (*p*FWEcorr > 0.10).

## Discussion

Many previous studies suggested that smelling food odors generates specific activation in the human brain as compared to non-food odors. In our fMRI study, involving three food and three non-food odorants matched in terms of intensity, pleasantness and trigeminal qualities, we attempted to verify this hypothesis. We observed that in our mixed sample of hungry and satiated participants, food odors generated significantly higher activation in the ACC (right and left), insula (right), and putamen (ventral striatum; right) than non-food odors. Among hungry subjects, regardless of odor type, we found significant activation in the VTA in response to olfactory stimulation.

We were particularly interested in activation of the reward circuit in response to food odors. Food is often considered to be a primary reward, necessary for human survival, and—as discussed in the introduction—different types of food-related stimuli activate brain reward circuits (Beaver et al., 2006; Stoeckel et al., 2008; Pursey et al., 2014). It has been hypothesized that the same applies to food odors. However, previous studies on this issue suffered from some methodological problems which we intended to resolve in the present paper. Our data confirm some of the previously published findings. First of all, we showed that food odors generated activation in putamen. This result is most probably due to the role in reward learning of this structure; putamen was consistently found to be involved in stimulus-action-reward association learning (Haruno and Kawato, 2005). Secondly, similar to a previous, mixed-design study (Jiang et al., 2015) and a study conducted among hungry subjects (Bragulat et al., 2010), we observed a large cluster of activated voxels in anterior cingulate. This finding is particularly interesting, since anterior cingulate cortex activity reflects, or perhaps regulates, the degree of reward anticipation (Shidara and Richmond, 2002). In addition, because of its functional connectivity to Nac/ventral striatum, anterior cingulate might be involved in reward-based decision making (Bush et al., 2002). Crucially, our study and previous research show this effect regardless of the participants' hunger/satiety status and various perceptual qualities of odorants. Thus, ACC seems to be a universal reward-related structure involved in specific food odor processing.

As for other elements of the reward circuit, in comparison with previous research (Bragulat et al., 2010; Frasnelli et al., 2015) we observed activation in VTA in response to both food- and non-food related stimuli, but only among hungry subjects. This effect might be due to enhanced reward sensitivity generated by hormonal changes related to hunger in these subjects, like e.g., increased ghrelin level (Malik et al., 2008). Thus, our data confirm the previously observed VTA activation in response to subjectively pleasant stimuli (Kühn and Gallinat, 2012; Simmons et al., 2014), but our results indicate that such an activation is generated only under specific conditions.

Food-related odorants also generated insular activation. Consistent with previous studies showing that the right hemisphere is of higher significance for the processing of olfactory stimuli as compared to the left one (Savic et al., 2000), we observed the insula to be activated on the right, but not left side of the brain. This effect is also similar to results in a study conducted among hungry subjects by Eiler et al. (2012). It could be hypothesized that insular activation in our case might be due to perceived sweetness, as our food odors were on average perceived as sweeter than the non-food odors. Generally, perceived sweetness of odors correlates with the magnitude of insular activation for food, but not for non-food odors, as reported by Veldhuizen et al. (2010). These authors suggested this finding indicates that some elements of taste cortex might also contribute to perception of odors associated with food. However, in our analyses we controlled for sweetness of the stimuli, so this difference between food and non-food odorants should not have had a major influence on the observed results.

### Limitations and future directions

The method of our study involved passive inhalation of the odorants. Although this is commonly applied in scientific studies assessing odor perception (Small et al., 2005; Bensafi et al., 2012; Iannilli et al., 2015; Sorokowska et al., 2016), active inhalation is how odors are typically experienced in everyday life. In future studies, it could be tested whether these two methods result in different perceptions of food related odors. In experimental designs involving active sniffing, potential confounds of sniff size could be mitigated by either measuring breathing activity throughout the experiment and controlling for any differences (Porter et al., 2005), or including active sniffing of a no odor condition and comparing odor conditions to that control stimulus.

A certain limitation of our study is that, similar to previous studies on food odor perception (e.g., Frasnelli et al., 2015), we used only pleasant (and potentially rewarding) odors. This issue is further highlighted by the activation of pallidum, insula and putamen in response to pooled food and non-food odors. For example, ventral pallidum is involved in food-related decision-making based on assessment of stimulus pleasantness (Simmons et al., 2014). It would be interesting to test whether the effects we found would also extend to unpalatable food and/or aversive non-food odors. Previous studies analyzing activation generated by unpleasant smells often involved food related odors (Royet et al., 2003), but they did not compare edible and non-edible odor sources. It is very interesting whether pertaining to the food category would be enough to overcome the effect of unpleasantness of odors and evoke food-related reward circuit activation. This would mean that the rewarding outcome of food ingestion might be more important than subjective hedonic value of food-associated olfactory stimuli.

## Conclusion

In conclusion, our data confirm the previously observed activation in the ACC and insula in response to food odors. As our stimuli were matched in terms of various perceptual qualities, this result suggests that edibility of an odor source generates specific activation in the human brain. However, more research is needed to determine the sources of inconsistent patterns of activations across other brain structures between our research and previous studies.

## Author contributions

AS, JW, and TH contributed to the conception and design of the work, AS and KS collected the data, KS, CH, and PH analyzed the data, and all authors interpreted the data for the work. AS, KS, CH, and PH drafted the work and JW and TH revising it critically for important intellectual content. All authors approved of the version to be published and agreed to be accountable for all aspects of the work in ensuring that questions related to the accuracy or integrity of any part of the work are appropriately investigated and resolved.

### Conflict of interest statement

The authors declare that the research was conducted in the absence of any commercial or financial relationships that could be construed as a potential conflict of interest.

## References

[B1] Beaver J. D. Lawrence A. D. van Ditzhuijzen J. Davis M. H. Woods A. Calder A. J. (2006). Individual differences in reward drive predict neural responses to images of food. J. Neurosci. 26, 5160–5166. 10.1523/JNEUROSCI.0350-06.2006 16687507 PMC6674259

[B2] Bensafi M. Iannilli E. Poncelet J. Seo H. S. Gerber J. Rouby C.. (2012). Dissociated representations of pleasant and unpleasant Olfacto-trigeminal mixtures: an fMRI study. PLoS ONE 7:e38358. 10.1371/journal.pone.0038358 22701631 PMC3373527

[B3] Berridge K. C. (1996). Food reward : brain substrates of wanting and liking. Neurosci. Biobehav. Rev. 20, 1–25. 10.1016/0149-7634(95)00033-B 8622814

[B4] Boesveldt S. Frasnelli J. Gordon A. R. Lundström J. N. (2010). The fish is bad: negative food odors elicit faster and more accurate reactions than other odors. Biol. Psychol. 84, 313–317. 10.1016/j.biopsycho.2010.03.006 20227457

[B5] Bragulat V. Dzemidzic M. Bruno C. Cox C. A. Talavage T. Considine R. V.. (2010). Food-related odor probes of brain reward circuits during hunger: a pilot fMRI study. Obesity (Silver Spring) 18, 1566–1571. 10.1038/oby.2010.57 20339365

[B6] Brett M. Anton J. Valabregue R. Poline J. (2002). Region of interest analysis using an SPM toolbox [abstract], in Proceedings of the 8th International Conference on Functional Mapping of the Human Brain (Sendai).

[B7] Bush G. Vogt B. A. Holmes J. Dale A. M. Greve D. Jenike M. A.. (2002). Dorsal anterior cingulate cortex: a role in reward-based decision making. Proc. Natl. Acad. Sci. U.S.A. 99, 523–528. 10.1073/pnas.012470999 11756669 PMC117593

[B8] DiLeone R. J. Taylor J. R. Picciotto M. R. (2012). The drive to eat: comparisons and distinctions between mechanisms of food reward and drug addiction. Nat. Neurosci. 15, 1330–1335. 10.1038/nn.3202 23007187 PMC3570269

[B9] Drewnowski A. (1998). Energy density, palatability, and satiety: implications for weight control. Nutr. Rev. 56, 347–353. 10.1111/j.1753-4887.1998.tb01677.x 9884582

[B10] Eiler W. J. A. Dzemidzic M. Case K. R. Considine R. V. Kareken D. A. (2012). Correlation between ventromedial prefrontal cortex activation to food aromas and cue-driven eating: an fMRI study. Chemosens. Percept. 5, 27–36. 10.1007/s12078-011-9112-6 25485031 PMC4255712

[B11] Fjaeldstad A. Fernandes H. M. Van Hartevelt T. J. Gleesborg C. Møller A. Ovesen T.. (2017). Brain fingerprints of olfaction: a novel structural method for assessing olfactory cortical networks in health and disease. Sci. Rep. 7:42534. 10.1038/srep42534 28195241 PMC5307346

[B12] Frasnelli J. Hummel C. Bojanowski V. Warr J. Gerber J. Hummel T. (2015). Food-related odors and the reward circuit: functional MRI. Chemosens. Percept. 8, 192–200. 10.1007/s12078-015-9193-8

[B13] Gottfried J. A. Zald D. H. (2005). On the scent of human olfactory orbitofrontal cortex: meta-analysis and comparison to non-human primates. Brain Res. Rev. 50, 287–304. 10.1016/j.brainresrev.2005.08.004 16213593

[B14] Haruno M. Kawato M. (2005). Different neural correlates of reward expectation and reward expectation error in the putamen and caudate nucleus during stimulus-action-reward association learning. J. Neurophysiol. 95, 948–959. 10.1152/jn.00382.2005 16192338

[B15] Hummel T. Kobal G. Gudziol H. Mackay-Sim A. (2007). Normative data for the 'Sniffin' Sticks' including tests of odor identification, odor discrimination, and olfactory thresholds: an upgrade based on a group of more than 3,000 subjects. Eur. Arch. Otorhinolaryngol. 264, 237–243. 10.1007/s00405-006-0173-0 17021776

[B16] Hummel T. Sekinger B. Wolf S. R. Pauli E. Kobal G. (1997). ‘Sniffin’ sticks': olfactory performance assessed by the combined testing of odour identification, odor discrimination and olfactory threshold. Chem. Senses 22, 39–52. 10.1093/chemse/22.1.39 9056084

[B17] Iannilli E. Sorokowska A. Zhigang Z. Hähner A. Warr J. Hummel T. (2015). Source localization of event-related brain activity elicited by food and nonfood odors. Neuroscience 289, 99–105. 10.1016/j.neuroscience.2014.12.044 25592427

[B18] Jiang T. Soussignan R. Schaal B. Royet J.-P. (2015). Reward for food odors: an fMRI study of liking and wanting as a function of metabolic state and BMI. Soc. Cogn. Affect. Neurosci. 10, 561–568. 10.1093/scan/nsu086 24948157 PMC4381239

[B19] Kareken D. A. Claus E. D. Sabri M. Dzemidzic M. Kosobud A. E. Radnovich A. J.. (2004). Alcohol-related olfactory cues activate the nucleus accumbens and ventral tegmental area in high-risk drinkers: preliminary findings. Alcohol. Clin. Exp. Res. 28, 550–557. 10.1097/01.ALC.0000122764.60626.AF 15100605

[B20] Kühn S. Gallinat J. (2012). The neural correlates of subjective pleasantness. Neuroimage 61, 289–294. 10.1016/j.neuroimage.2012.02.065 22406357

[B21] Lieberman L. S. (2003). Dietary, evolutionary, and modernizing influences on the prevalence of type 2 diabetes. Annu. Rev. Nutr. 23, 345–377. 10.1146/annurev.nutr.23.011702.073212 12651966

[B22] Maldjian J. A. Laurienti P. J. Kraft R. A. Burdette J. H. (2003). An automated method for neuroanatomic and cytoarchitectonic atlas-based interrogation of fMRI data sets. Neuroimage 19, 1233–1239. 10.1016/S1053-8119(03)00169-1 12880848

[B23] Malik S. McGlone F. Bedrossian D. Dagher A. (2008). Ghrelin modulates brain activity in areas that control appetitive behavior. Cell Metab. 7, 400–409. 10.1016/j.cmet.2008.03.007 18460331

[B24] Oldfield R. C. (1971). The assessment and analysis of handedness: the Edinburgh inventory. Neuropsychologia 9, 97–113. 10.1016/0028-3932(71)90067-4 5146491

[B25] Porter J. Anand T. Johnson B. Khan R. M. Sobel N. (2005). Brain Mechanisms for Extracting Spatial Information from Smell. Neuron 47, 581–592. 10.1016/j.neuron.2005.06.028 16102540

[B26] Pursey K. M. Stanwell P. Callister R. J. Brain K. Collins C. E. Burrows T. L. (2014). Neural responses to visual food cues according to weight status: a systematic review of functional magnetic resonance imaging studies. Front. Nutr. 1:7. 10.3389/fnut.2014.00007 25988110 PMC4428493

[B27] Rolls E. T. (2015). Taste, olfactory, and food reward value processing in the brain. Prog. Neurobiol. 127–128, 64–90. 10.1016/j.pneurobio.2015.03.002 25812933

[B28] Royet J.-P. Plailly J. Delon-Martin C. Kareken D. A. Segebarth C. (2003). fMRI of emotional responses to odors: influence of hedonic valence and judgment, handedness, and gender. Neuroimage 20, 713–728. 10.1016/S1053-8119(03)00388-4 14568446

[B29] Savic I. Gulyas B. Larsson M. Roland P. (2000). Olfactory functions are mediated by parallel and hierarchical processing. Neuron 26, 735–745. 10.1016/S0896-6273(00)81209-X 10896168

[B30] Schifferstein H. N. J. Verlegh P. W. J. (1996). The role of congruency and pleasantness in odor-induced taste enhancement. Acta Psychol. (Amst). 94, 87–105. 10.1016/0001-6918(95)00040-2 8885712

[B31] Seubert J. Freiherr J. Djordjevic J. Lundström J. N. (2013). Statistical localization of human olfactory cortex. Neuroimage 66, 333–342. 10.1016/j.neuroimage.2012.10.030 23103688

[B32] Shidara M. Richmond B. J. (2002). Anterior cingulate: single neuronal signals related to degree of reward expectancy. Science (80) 296, 1709–1711. 10.1126/science.1069504 12040201

[B33] Simmons W. K. Rapuano K. M. Ingeholm J. E. Avery J. Kallman S. Hall K. D.. (2014). The ventral pallidum and orbitofrontal cortex support food pleasantness inferences. Brain Struct. Funct. 219, 473–483. 10.1007/s00429-013-0511-0 23397317 PMC3676475

[B34] Small D. M. Gerber J. C. Mak Y. E. Hummel T. (2005). Differential neural responses evoked by orthonasal versus retronasal odorant perception in humans. Neuron 47, 593–605. 10.1016/j.neuron.2005.07.022 16102541

[B35] Sommer J. U. Maboshe W. Griebe M. Heiser C. Hörmann K. Stuck B. A.. (2012). A mobile olfactometer for fMRI-studies. J. Neurosci. Methods 209, 189–194. 10.1016/j.jneumeth.2012.05.026 22683953

[B36] Sorokowska A. Negoias S. Härtwig S. Gerber J. Iannilli E. Warr J.. (2016). Differences in the central-nervous processing of olfactory stimuli according to their hedonic and arousal characteristics. Neuroscience 324, 62–68. 10.1016/j.neuroscience.2016.03.008 26968764

[B37] Stevenson R. J. (2010). An initial evaluation of the functions of human olfaction. Chem. Senses 35, 3–20. 10.1093/chemse/bjp083 19942579

[B38] Stice E. Figlewicz D. P. Gosnell B. A. Levine A. S. Pratt W. E. (2013). The contribution of brain reward circuits to the obesity epidemic. Neurosci. Biobehav. Rev. 37, 2047–2058. 10.1016/j.neubiorev.2012.12.001 23237885 PMC3604128

[B39] Stoeckel L. E. Weller R. E. Cook E. W. III. Twieg D. B. Knowlton R. C. Cox J. E. (2008). Widespread reward-system activation in obese women in response to pictures of high-calorie foods. Neuroimage 41, 636–647. 10.1016/j.neuroimage.2008.02.031 18413289

[B40] Tzourio-Mazoyer N. Landeau B. Papathanassiou D. Crivello F. Etard O. Delcroix N.. (2002). Automated anatomical labeling of activations in SPM using a macroscopic anatomical parcellation of the MNI MRI single-subject brain. Neuroimage 15, 273–289. 10.1006/nimg.2001.0978 11771995

[B41] Veldhuizen M. G. Nachtigal D. Teulings L. Gitelman D. R. Small D. M. (2010). The insular taste cortex contributes to odor quality coding. Front. Hum. Neurosci. 4:58. 10.3389/fnhum.2010.00058 20700500 PMC2917218

